# The transcriptomic signature of respiratory sensitizers using an alveolar model

**DOI:** 10.1007/s10565-024-09860-x

**Published:** 2024-04-08

**Authors:** Matthew Gibb, James Y. Liu, Christie M. Sayes

**Affiliations:** 1 Institute of Biomedical Studies (BMS), Baylor University, Waco, TX 76798-7266 USA; 2 Department of Environmental Science (ENV), Baylor University, One Bear Place #97266, Waco, TX 76798-7266 USA

**Keywords:** Respiratory sensitization, Genetic expression, Immunotoxicology, RNA-sequencing, Occupational health

## Abstract

Environmental contaminants are ubiquitous in the air we breathe and can potentially cause adverse immunological outcomes such as respiratory sensitization, a type of immune-driven allergic response in the lungs. Wood dust, latex, pet dander, oils, fragrances, paints, and glues have all been implicated as possible respiratory sensitizers. With the increased incidence of exposure to chemical mixtures and the rapid production of novel materials, it is paramount that testing regimes accounting for sensitization are incorporated into development cycles. However, no validated assay exists that is universally accepted to measure a substance’s respiratory sensitizing potential. The lungs comprise various cell types and regions where sensitization can occur, with the gas-exchange interface being especially important due to implications for overall lung function. As such, an assay that can mimic the alveolar compartment and assess sensitization would be an important advance for inhalation toxicology. Some such models are under development, but in-depth transcriptomic analyses have yet to be reported. Understanding the transcriptome after sensitizer exposure would greatly advance hazard assessment and sustainability. We tested two known sensitizers (*i.e.,* isophorone diisocyanate and ethylenediamine) and two known non-sensitizers (*i.e.,* chlorobenzene and dimethylformamide). RNA sequencing was performed in our in vitro alveolar model, consisting of a 3D co-culture of epithelial, macrophage, and dendritic cells. Sensitizers were readily distinguishable from non-sensitizers by principal component analysis. However, few differentially regulated genes were common across all pair-wise comparisons (*i.e.,* upregulation of genes *SOX9*, *UACA*, *CCDC88A*, *FOSL1*, *KIF20B*). While the model utilized in this study can differentiate the sensitizers from the non-sensitizers tested, further studies will be required to robustly identify critical pathways inducing respiratory sensitization.

**Graphical Abstract:**

Graphical headlines/headlights

- Pollutants may trigger lung allergies, but no universal method measures respiratory sensitization potential.
- In vitro systems can detect respiratory sensitizers, aiding in anticipating and reducing the risks of new materials.
- Sensitizers and non-sensitizers can be distinguished through transcriptome investigation.
- The sensitizers tested induced cell differentiation and proliferation pathways while inhibiting immune defense and functionality.

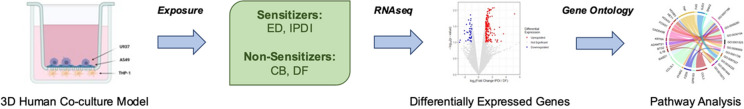

**Supplementary Information:**

The online version contains supplementary material available at 10.1007/s10565-024-09860-x.

## Introduction

The lungs are continuously exposed to exogenous materials carried by inhaled air including aerosolized chemicals, particulate matter, and microbes (Paur et al. 2011; Thompson 2018; Mack et al. 2019; Cao et al. 2021). Because of the large surface area and central role in respiration, the lungs are critically important for understanding how inhaled air can affect and potentially alter the immune responses, especially at the gas-exchange interface of the alveoli (Guillot et al. 2013; Glencross et al. 2020; Invernizzi et al. 2020). Among the potential adverse immunological outcomes in the lungs is ‘respiratory sensitization’ (North et al. 2016; Sullivan et al. 2017; Gibb and Sayes 2022). The sensitization process includes an initial exposure (induction phase), where immune cells are primed for an exacerbated response, and a secondary exposure (elicitation phase), where the exacerbated response manifests, and allergic reactions occur (Kwangsukstith and Maibach 1995; Michaels 2016). The ability to predict if a substance induces sensitization, termed ‘sensitizer’, without elicitation has recently been studied using in vitro and high-throughput approaches.

There are four main types of hypersensitivity, which are numerically labeled (Meth and Sperber 2006). Types I, II, and III are considered immediate reactions within 24 h after initial exposure (Vaillant et al. 2023). Type IV hypersensitivity can be described as cell-mediated and usually occurs after subsequent exposure to an allergen. Some low molecular weight chemicals, such as diisocyanates and ethylenediamine, are known respiratory allergens that lead to Type IV reactions, which include the recruitment of leukocytes (*e.g.,* T-cells), leading to long-term chronic conditions (Pronk et al. 2007; Forreryd et al. 2015). Methods to identify sensitization and hypersensitivity types before exposure to hazardous substances are lacking. In vitro models are continuously being developed, tested, and improved to assess respiratory sensitizing potential (Biaglow et al. 1978; Verstraelen et al. 2008; Forreryd et al. 2015; Hermanns et al. 2015; Chary et al. 2019; Gibb and Sayes 2022). Rodent testing, specifically the inhalation-based local lymph node assay (LLNA), is currently the only validated method with some level of acceptance. Still, it is resource intensive, not readily suited to high-throughput screening, and subject to ethical controversy, and the results do not always translate accurately to humans (Hackam and Redelmeier 2006; Bracken 2009; Hoymann 2012; Thew 2012; Stoccoro et al. 2013; Fröhlich and Salar-Behzadi 2014). In vitro studies using human-derived cells may allow for cost-effective rapid assessment, with fewer confounders factors introduced by a dynamic in vivo organism, as well as the assessment of species-specific responses.

Assessing the potential of chemicals or particles to induce sensitization is challenging, as sensitization induces heterogeneous outcomes, and immune responses vary widely across individuals and populations. Once chemicals are found to induce sensitization, they are thoroughly investigated, and lists are regularly compiled to track known sensitizers for dermal and respiratory target organ systems. Studies have shown that inhaled diisocyanates and other low molecular weight chemicals, such as ethylenediamine, produce hypersensitivity in the lungs (Charles et al. 1976; Karol et al. 1980; Stadler and Karol 1984; Karol and Dean 1986; Venables 1989; Arts et al. 1998; Pauluhn 2003; Jose and Craig 2016). Diisocyanates are used by consumers and occupational workers, leading to inhalation exposure of vapors or aerosols. Ethylenediamine is found in agrochemicals, pharmaceuticals, and residential applications (Lam and Chan-Yeung 1980; Nakazawa and Matsui 1990; Sadekar et al. 2021). For this study, we chose a representative diisocyanate, isophorone diisocyanate (IPDI), and ethylenediamine (ED) as positive controls for respiratory sensitization. Chlorobenzene (CB) and dimethylformamide (DF) were negative controls for sensitization, but both are relevant chemicals for occupational inhalation exposure (Yoshida et al. 1986; Grammer et al. 1988; Redlich et al. 1988; Feoktistova et al. 1989; Vandenplas et al. 1993; Murata and Aono 1998; Kim and Kim 2011; Laborde-Castérot et al. 2012; Forreryd et al. 2015; Mowitz et al. 2016; Park et al. 2016; Antoniou et al. 2020).

Our previous study has shown that a simplified, architecturally relevant alveolar model can identify biomarkers associated with respiratory sensitization (Gibb and Sayes 2022). In that work, human lung and immune cells were exposed to the known pulmonary sensitizer isophorone diisocyanate (IPDI) and controls, investigating endpoints for oxidative stress, cytostructural changes, cell surface morphology, and cytokines. An in-depth gene expression analysis compared sensitizers to non-sensitizers to continue this work. Crucially, the transcriptome of dendritic cells was differentiated from that of macrophage and epithelial cells. Dendritic cells are known to upregulate migratory markers and travel to local lymph nodes during maturation, leading to inflammatory responses such as sensitization (Cavanagh and Von Andrian 2002; Angeli and Randolph 2006; Hilligan and Ronchese 2020; Liu et al. 2021; Redondo-Urzainqui et al. 2023; Mizoguchi et al. 2023). By examining the perturbations in the transcriptome due to known sensitizers and comparing them to those induced by known non-sensitizers, it is possible to gain greater insight into how respiratory sensitization occurs and contribute to advancing models toward high throughput screening of sensitizing potential.

## Materials and methods

### Cell culture

Epithelial cells (A549) and macrophages (differentiated U937) were grown in complete RPMI (cRPMI) 1640 (Thermo Fisher Scientific Inc. Waltham, MA, USA) with 10% FBS and 1% penicillin–streptomycin. Dendritic cells (differentiated THP-1) were cultured in cRPMI 1640 supplemented 2-mercaptoethanol at a final concentration of 0.05 mM. All cells were maintained at 37 °C in a humidified 5% and CO_2_ atmosphere.

### Macrophage differentiation

Monocytes (U937) were differentiated into macrophages after incubation with 100 ng/mL phorbol 12-myristate-13-acetate (PMA) for 24 to 48 h as previously described (Prasad et al. 2020). The cells were washed twice in sterile 1X PBS and fresh media was then added. Cells were then rested in a 37 °C humidified incubator at 5% CO_2_ atmosphere for 72 h before use. The adherent cells were dissociated using trypsin. Following trypsinization, the dissociated cells were resuspended, counted, and plated according to use.

### Dendritic cell differentiation

Monocytes (THP-1) were differentiated into dendritic cells via centrifugation and resuspension at 2 × 10^5^ cells/mL. Cells were cultured in a serum-free medium supplemented with rhIL-4 (200 ng/mL), rhGM-CSF (100 ng/mL), rhTNFa (20 ng/mL), and 200 ng/mL ionomycin. All cytokines were purchased from Thermo Fisher Scientific. Cells were cultured for 48 h in a humidified incubator at 37 °C and 5% CO_2_ and then used for experiments.

### Model description, assembly, and maintenance

**Fig. 1:**
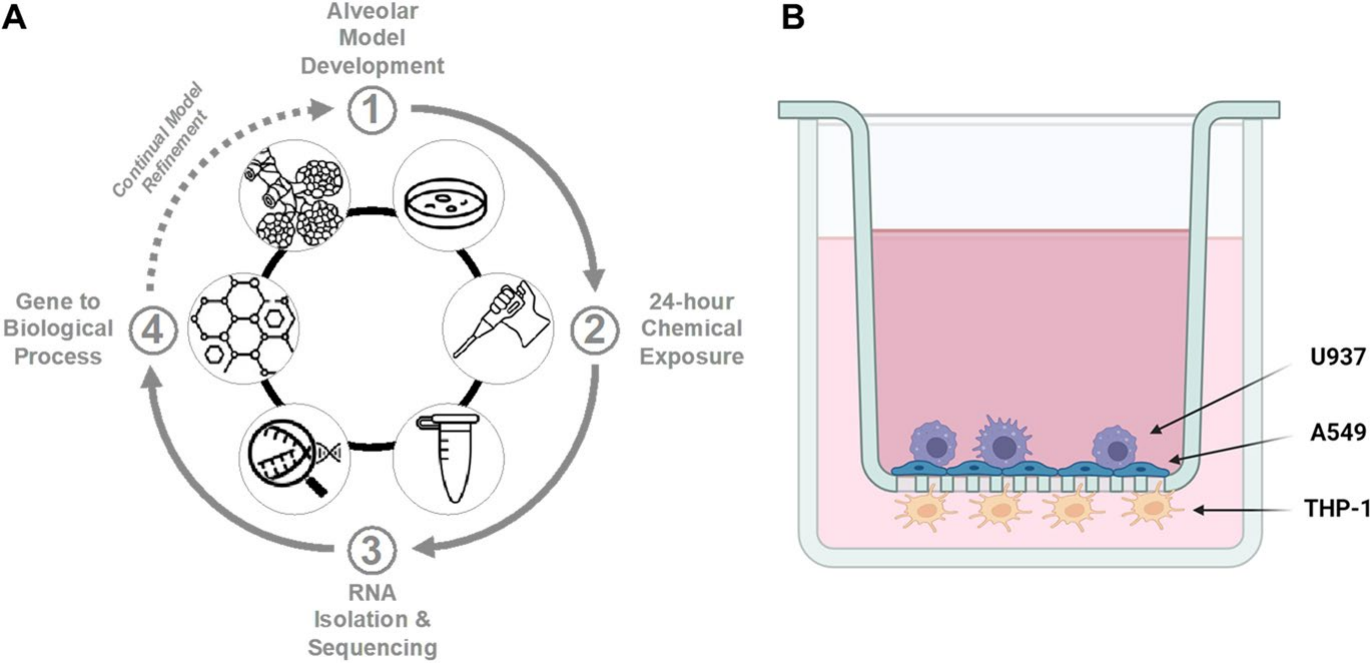
Depiction of study design and model system. **A** Alveolar model development (1) for testing respiratory sensitization (2), subsequent RNA-Sequencing analysis (3), and comparison to the published literature (4). This cycle enables model refinement and subsequent studies designed to assess the sensitizing potential of unknown materials rapidly. **B** The alveolar coculture model used in this study consisted of epithelial (A549) and macrophage (U937) cells on the apical side and dendritic (THP-1) cells on the basal side

Cells were plated as previously described (Lehmann et al. 2011; Drasler et al. 2020; Sayes and Singal 2021; Gibb and Sayes 2022). Epithelial cells were added to 12-well plates fitted with polyethylene terephthalate (PET) Transwell® membranes (Corning, Tewksbury, MA, USA) at 28 × 10^4^ cells/cm^2^ seeding density. Cells were allowed to adhere for 3 days or until a confluent monolayer was formed. The media was removed, and the inserts were inverted and placed into sterile glass Petri dishes. Dendritic cells (*i.e.,* differentiated THP-1 monocytes) were resuspended in 500 μL of complete media and plated on the basal surface of the membrane at 7 × 10^4^ cells/cm^2^ and allowed to adhere for 4 h. After adherence, excess media was removed, inserts were reverted into the well plate, and 1 mL of cRPMI 1640 with 2-mercaptoethanol was added to the basolateral chamber. Macrophages (*i.e.,* differentiated U937 monocytes) were added at a 1:9 ratio of U937:A549 in cRPMI (Pollmächer and Figge 2014). The in vitro alveolar cell culture model was completed (Fig. 1B) after replenishing media to a total volume of 500 μL. The model was then placed in a 37 °C humidified incubator at 5% CO_2_ atmosphere for 24 h before chemical exposure to sensitizers and non-sensitizers.

### Chemical exposures

All exposure chemicals were added to the apical compartment of the Transwell® membrane. Isophorone diisocyanate (IPDI, Thermo Fisher Scientific, Inc.) was added at 25 μM, and ethylenediamine (ED, Thermo Fisher Scientific, Inc.) was added at 500 μM as a positive control for sensitization (Forreryd et al. 2015); (Lam and Chan-Yeung 1980); (Stadler and Karol 1984; Nakazawa and Matsui 1990; Vandenplas et al. 1993). Chlorobenzene (CB, Thermo Fisher Scientific, Inc.) was added at 98 μM, and dimethylformamide (DF, Thermo Fisher Scientific, Inc.) was added at 500 μM as negative controls/non-sensitizers (Willhite and Book 1990; Forreryd et al. 2015). Dimethyl sulfoxide (DMSO) was used as a vehicle for the solubilization of IPDI and CB. The post-exposure period was 24 h to assess the early genetic expression of respiratory sensitizing potential. Sets of three wells each were treated with vehicle control or chemical exposures for 24 h, followed by RNA extraction and subsequent analysis.

### RNA extraction

RNA extraction and sequencing were performed at Azenta Life Sciences (South Plainfield, NJ, USA). Total RNA from frozen pellets was extracted using the Qiagen RNeasy Plus Universal mini kit (Qiagen, Hilden, Germany). RNA was quantified via Qubit 2.0 Fluorometer (Life Technologies, Carlsbad, CA, USA); integrity was checked using Agilent TapeStation 4200 (Agilent Technologies, Palo Alto, CA, USA); and sequencing libraries were prepared using the NEBNext Ultra II RNA Library Prep for Illumina (NEB, Ipswich, MA, USA). mRNAs were enriched with Oligod(T) beads and fragmented for 15 min at 94 °C. First-strand and second-strand cDNA were synthesized, fragments were repaired and adenylated at 3’ ends, and universal adapters were ligated to cDNA fragments, followed by index addition and library enrichment by PCR. The libraries were validated on the Agilent TapeStation (Agilent Technologies) and quantified by using Qubit 2.0 Fluorometer (Invitrogen) as well as by quantitative PCR (KAPA Biosystems, Wilmington, MA, USA).

The sequencing libraries were clustered on a lane of a HiSeq flow cell. The samples were sequenced using a 2 × 150 bp Paired-End (PE) configuration. The software conducted image analysis and base calling. Raw sequence data (.bcl files) generated by the sequencer were converted into fastq files and de-multiplexed using Illumina's bcl2fastq 2.17 software. One mismatch was allowed for index sequence identification.

### Data analysis

After investigating the quality of the raw data, sequence reads were trimmed to remove adapter sequences and mapped to the reference genome (ENSEMBL; STAR aligner v.2.5.2b). BAM files were generated, and unique gene hit counts were calculated using feature counts (Subread package v.1.5.2). Unique reads that fell within exon regions were counted.

After extraction of gene hit counts, downstream analysis was conducted using R statistical software. Log-twofold-change (L2FC) was calculated for all treatments normalized to untreated controls and for all pairs of sensitizers normalized to non-sensitizers. The Wald test *p*-value assessed the significance between sensitizer and non-sensitizer pairs. L2FC values > 1 in magnitude with *p*-values < 0.05 were marked as differentially expressed (full tables available in supplemental information). The top 40 genes ranked by L2FC for each pair-wise comparison were plotted in heatmaps, while volcano plots were used to visualize overall differential expression. Principal component analysis (PCA) was used to visualize the distinct groupings of sensitizers and non-sensitizers by variance for the two compartments. Differentially expressed genes from the aggregate of each compartment were fed into the Gene Ontology (GO) database, and the most common features were collected for the biological process. The top 10 terms associated with differentially expressed genes (sensitizer against non-sensitizer) with a minimum hierarchical distance of 3 from the root (i.e. biological process) were reported as chord diagrams. Analysis and visualization employed the packages gplots, ggplot2, viridis, dplyr, tidyverse, GO.db, annotate, org.Hs.eg.db, and circlize.

## Results

Model development (Fig. 1A) is based on in vivo alveolar architecture where epithelial and macrophage cells are in the apical chamber of the Transwell^®^ with dendritic cells in the basal chamber (Fig. 1B). Panels in the figure show this model's possible continual flow of input and data readout. Panel 2 depicts exposure to xenobiotics (chemical or particulate) that can be added to the model. Panel 3 depicts data analysis with this study highlighting RNA-Seq. Panel 4 depicts comparisons from Panel 3 to known outcomes in human populations if available in the literature (case studies, population-level studies, etc*.*). All analyses were normalized to untreated controls before comparisons between sensitizers and non-sensitizers.

### Gene expression of epithelial and macrophage cells

Exposure to sensitizer ED compared to non-sensitizer CB revealed 15 upregulated and 17 downregulated genes (Supplemental Table 1). Exposure to ED to non-sensitizer dimethylformamide (DF) revealed 38 upregulated and 21 downregulated genes (Supplemental Table 2). Exposure to sensitizer IPDI compared to CB revealed 15 upregulated and 22 downregulated genes (Supplemental Table 3). Exposure to IPDI compared to DF revealed 38 upregulated and 29 downregulated genes (Supplemental Table 4).

When comparing the sensitizer ED to both non-sensitizers CB and DF, there were two upregulated genes (*ALPK2*, *KRT6A*) and two downregulated genes (*CD101*, *P2RX1*) in common. Comparing the sensitizer IPDI to both non-sensitizers CB and DF revealed 5 upregulated genes (*RASD1*, *DUSP2*, *ADAMTS1*, *AL731577.2*, *FZD9*) and 5 downregulated genes (*MT-TE*, *DLEU7*, *ZNF230*, *ZNF416*, *RAB44*).When comparing sensitizers ED and IPDI to the non-sensitizer CB, there were no upregulated genes and 5 downregulated genes (*SNAI3*, *IFITM1*, *ZNF2*, *ARL11*, *TLE6*) in common. Comparing both sensitizers to the non-sensitizer DF revealed 5 upregulated genes (*HERC2P2*, *HEY1*, *GADD45B*, *MREG*, *HOXA3*) and 4 downregulated genes (*RN7SL3*, *TMEM266*, *FCGR2B*, *RGS18*) in common. Two upregulated genes were common between sensitizers ED and IPDI compared to both non-sensitizers CB and DF (*CCL3L1*, *GJD3*).

### Gene expression of dendritic cells

Exposure to sensitizer ED compared to non-sensitizer CB revealed 65 upregulated genes and 14 downregulated genes (Supplemental Table 5). Compared to the non-sensitizer DF, exposure to ED showed 305 upregulated genes and 30 downregulated genes (Supplemental Table 6). Exposure to sensitizer IPDI compared to CB showed 34 upregulated and 34 downregulated genes (Supplemental Table 7). Compared to DF, exposure to IPDI showed 207 upregulated and 43 downregulated genes (Supplemental Table 8).

Sensitizer ED, compared to both non-sensitizers CB and DF, showed 18 upregulated genes (*AL139099.4*, *HIST1H4A*, *SCARNA12*, *HIST2H2AB*, *HIST1H1E*, *HIST1H1B*, *SCARNA7*, *HIST2H3D*, *HIST1H2BE*, *SCARF2*, *LINC00294*, *HIST1H4E*, *HIST2H2BF*, *GCC2*, *CEP350*, *PIBF1*, *IGF2BP2*, *ZC3H13*) and two downregulated genes (*HLA*, *UQCRHL*) in common. Sensitizer IPDI compared to both non-sensitizers CB and DF showed 10 upregulated genes (*LRRC37A3*, *ENSG00000272579*, *CEP152*, *CEMIP*, *AXIN2*, *DNAJC2*, *NR2F1*, *NSRP1*, *GATA2*, *KIF21A*) and 9 downregulated genes (*PLAG1*, *FAM212A*, *ZNF416*, *ZNF449*, *ZNF671*, *ZNF230*, *FCGR2B*, *BATF2*, *PARS2*) in common. Both sensitizers ED and IPDI, compared to both non-sensitizers CB and DF, showed 7 upregulated genes (*EGR3*, *IL10*, *NFIB*, *NR4A3*, *LMTK3*, *GEM*, *RND1*) and 4 downregulated genes (*ZKSCAN4*, *P2RY12*, *CCR5*, *ZNF250*). Compared to non-sensitizer DF, both sensitizers ED and IPDI showed 11 upregulated and 129 downregulated genes. There were no common downregulated genes between the sensitizers and non-sensitizers. Common to both sensitizers ED and IPDI compared to both non-sensitizers CB and ED were the upregulated genes *SOX9*, *UACA*, *CCDC88A*, *FOSL1*, and *KIF20B*.

**Fig. 2:**
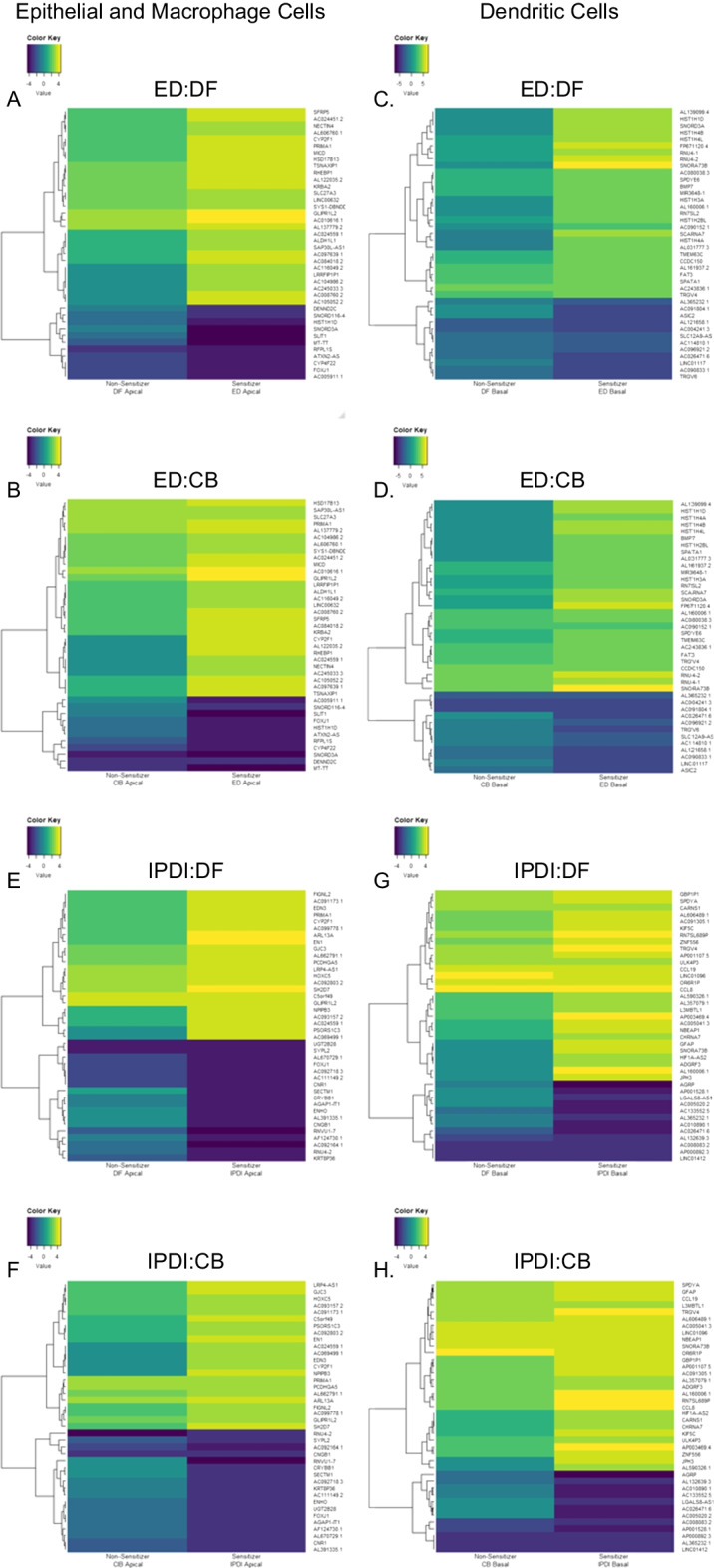
A bi-clustering heatmap was used to visualize the expression profile of the top 40 differentially expressed genes sorted by their *p*-value by plotting their log2 transformed expression values in samples. Acronyms: ethylenediamine (ED), chlorobenzene (CB), dimethylformamide (DF), and isophorone diisocyanate (IPDI)

**Fig. 3:**
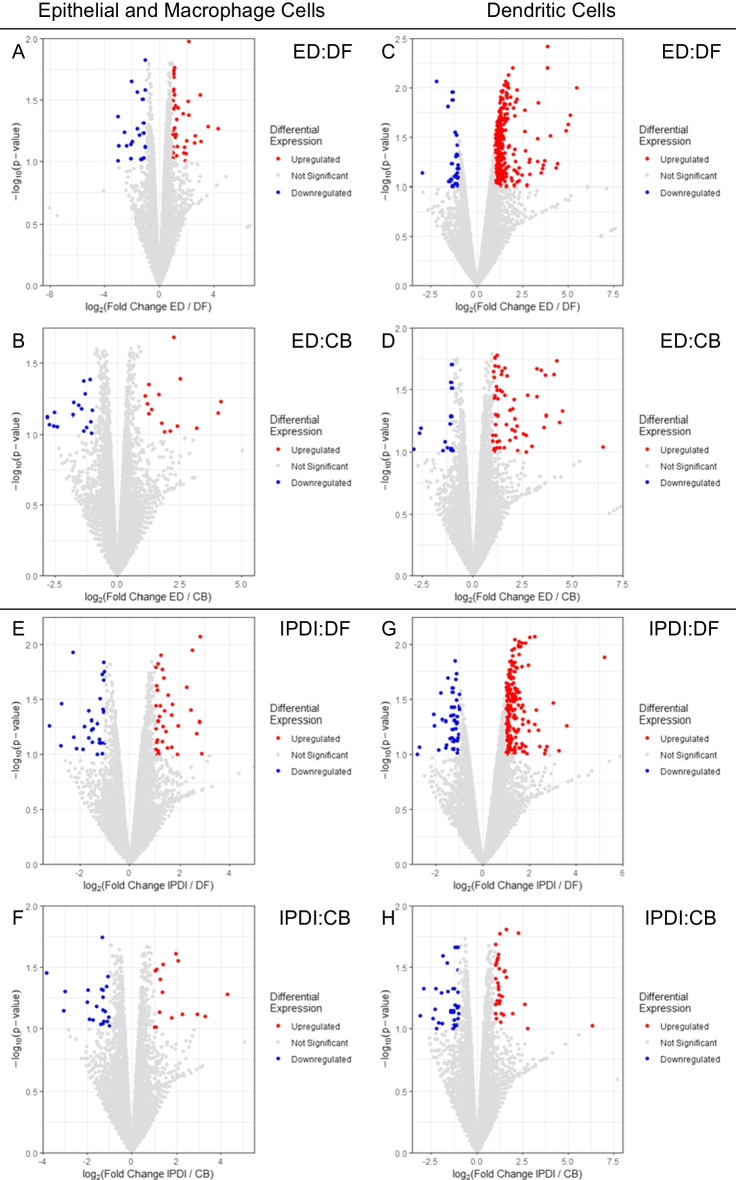
Global transcriptional change visualized via volcano plots. Each data point in the scatter plot represents a gene. Each gene’s log2 fold change is represented on the x-axis, and the log10 of its *p*-value is on the y-axis. Genes with a *p*-value less than 0.1 and a log2 fold change greater than 1 are indicated by red dots (representing up-regulation). Genes with a *p*-value less than 0.1 and a log2 fold change less than -1 are indicated by blue dots (representing down-regulation). Panels **A**–**D** represent the sensitizer ethylenediamine (ED) where **A** and **B** are macrophage and epithelial cells, and **C** and **D** are dendritic cells. Panels **E**–**H** represent sensitizer isophorone diisocyanate (IPDI), where **E** and **F** are macrophage and epithelial cells, and **G** and **H** are dendritic cells

Heatmaps (Fig. 2) and volcano plots (Fig. 3) show each comparison's significantly upregulated and downregulated genes. Data in each figure are limited to the top 40 differentially expressed genes, and volcano plots show all genes with significantly upregulated genes in red and significantly downregulated genes in blue.

**Fig. 4:**
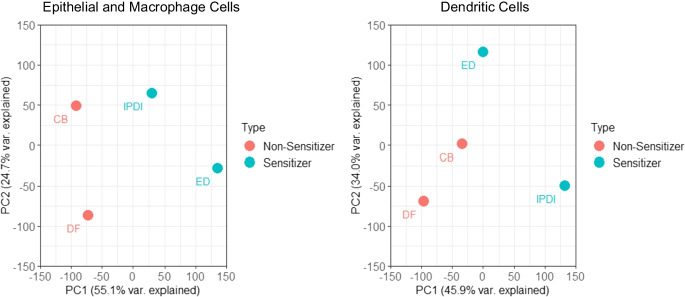
The samples were projected to a 2D plane spanned by their first two principal components. The x-axis is the direction that explains the most variance, and the y-axis is the second most. The percentage of the total variance per direction is shown on the label. Acronyms: ethylenediamine (ED), chlorobenzene (CB), dimethylformamide (DF), and isophorone diisocyanate (IPDI)

Figure 4 shows principal component analysis (PCA) in a 2D plane comparing the first two principal components using the default function prcomp in R and the ggplot2 package. As the figure shows, there is a clear delineation between sensitizers and non-sensitizers for both apical (epithelial and macrophage) and basal (dendritic) cells. Both sensitizers, ethylenediamine (ED) and isophorone diisocyanate (IPDI), were compared to two known non-sensitizers, chlorobenzene (CB) and dimethylformamide (DF). Analysis was performed individually on apical and basal compartments.

**Fig. 5:**
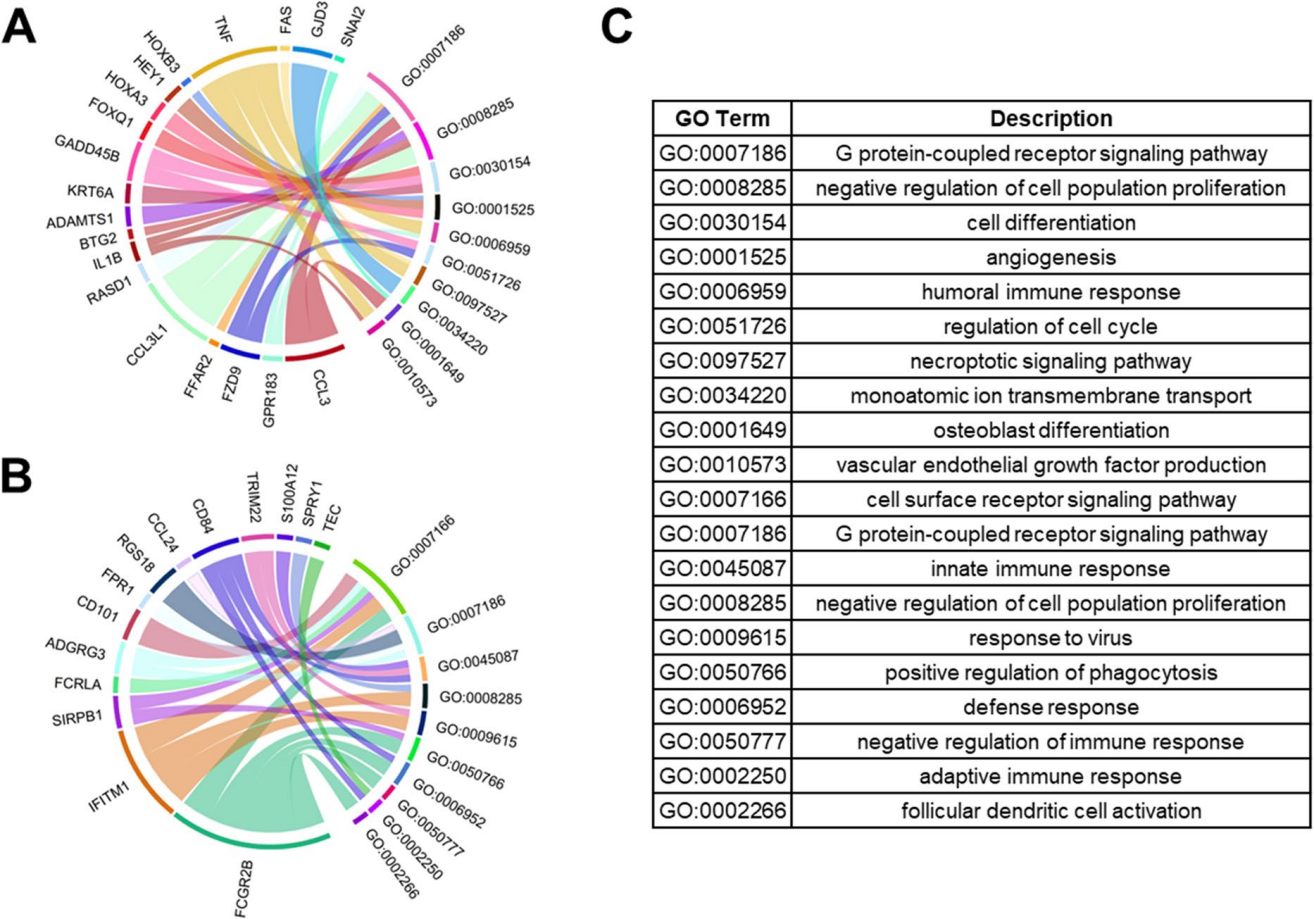
Chord diagrams depict associations between differentially expressed genes for apical cells and GO terms for biological processes (minimum distance of 3 from root). **A** The top 10 GO terms for upregulated genes. **B** The top 10 GO terms for downregulated genes. **C** Descriptions of the GO terms

**Fig. 6:**
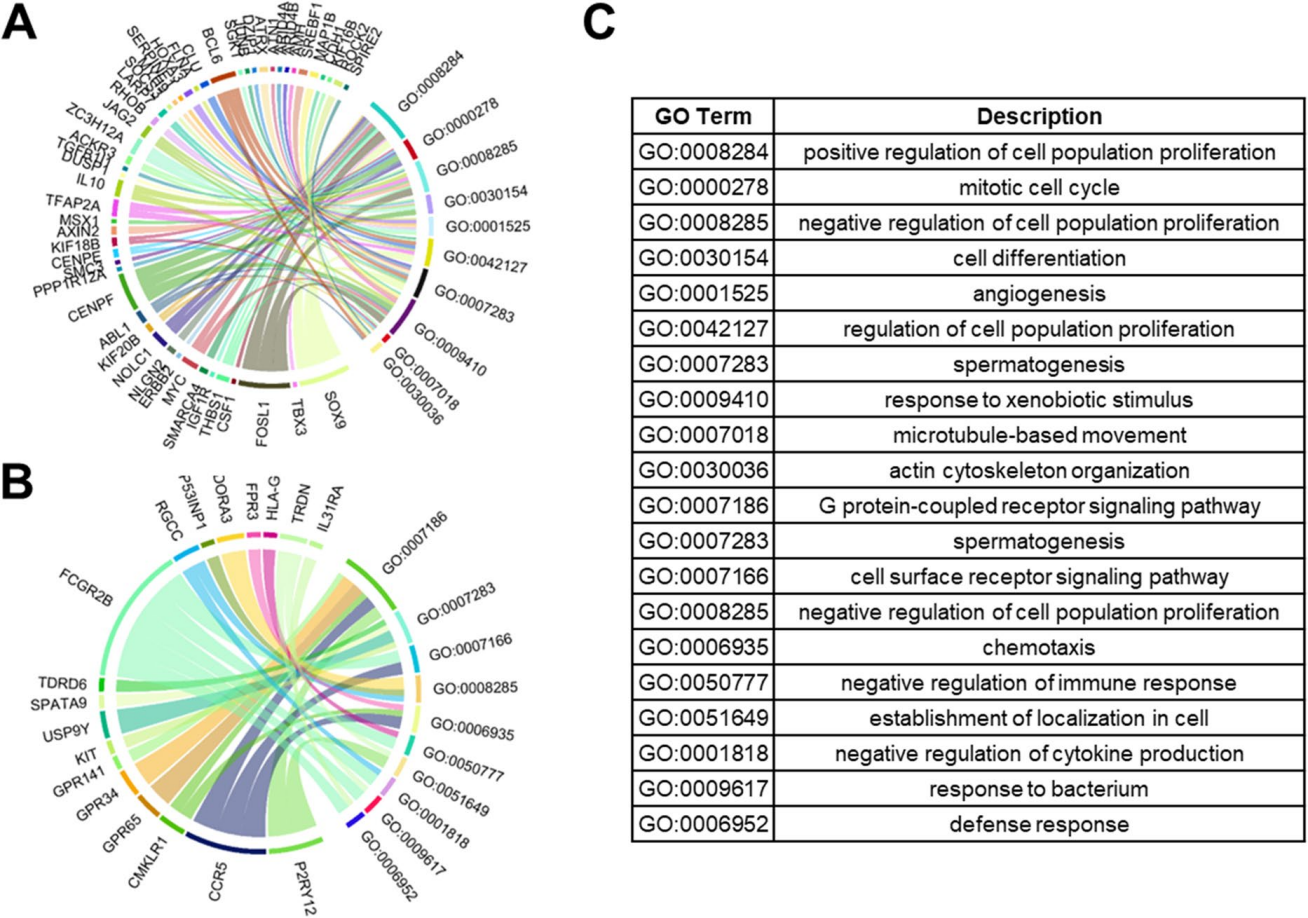
Chord diagrams depicting the associations between differentially expressed genes for basal cells and GO terms for biological process (minimum distance of 3 from root). **A** The top 10 GO terms for upregulated genes. **B** The top 10 GO terms for downregulated genes. **C** Descriptions of the GO terms

Significantly differentially regulated genes were cross-referenced against the gene ontology database as described in the methods section for analysis of perturbed biological processes. Matches between genes and GO terms were ordered by frequency of association, and the top 10 GO terms were reported as chord diagrams (Figs. 5 and 6).

To compare sensitizers to non-sensitizers, the associations were separated by cell compartment (apical/basal) and regulation (up/down). Among apical cells, pathways in upregulated genes are heavily associated with *receptor signaling, cell cycling, and humoral responses* (Fig. 5A). Downregulated genes from apical cells were most associated with *receptor signaling, innate and adaptive immune responses, and phagocytosis* (Fig. 5B).

For the basal DCs, pathways from upregulated genes were heavily associated with *cell differentiation, actin cytoskeleton organization, microtubule movement, and response to xenobiotic stimulus* (Fig. 6A). Pathways from downregulated genes in basal DCs were heavily associated with *receptor signaling, chemotaxis, negative regulation of immune response, negative regulation of cytokine production, and defense response* (Fig. 6B).

## Discussion

In a steady state, the lumen of the alveolus typically contains alveolar macrophages, epithelial cells, and secretion fluids (*e.g.,* lung surfactant) (Bissonnette et al. 2020). Alveolar macrophages (AM) dampen inflammatory responses in both steady state and perturbed conditions unless a continuous stimulus elicits a long-term immune response such as allergy or in response to pathogenic assault (*e.g.,* influenza or tuberculosis) (Bissonnette et al. 2020; Clements and Idoyaga 2021; Gibb and Sayes 2022). AMs suppress the inflammatory responses in dendritic cells (DCs) (Sibille and Reynolds 1990; Bedoret et al. 2009; Toussaint et al. 2013). However, respiratory sensitizers can overcome this suppression, leading to activation of DCs where recognition, capture, and processing of antigens occurs, causing upregulation of activation markers (*i.e.*, CD40, CD80, or CD86) along with migratory receptors (*e.g.,* CCR7). These markers instruct DCs to travel to local lymph nodes and initiate a cascade of events where long-term immune cells (*e.g.,* T and B cells) are formed, primed, and cause allergic reactions at the respiratory interface (Holt et al. 1994; Abbas et al. 2010; Forreryd et al. 2015). Early detection of these responses is critical to preventing poor health outcomes associated with the introduction of novel xenobiotics as well as naturally produced materials that may enter the environment or consumer and commercial products.

Sensitization in dermal tissue is well-established, with several universal protocols to measure sensitizing potentiality at multiple stages (Botham et al. 1991; Ashikaga et al. 2006). Unlike the skin, however, no universal protocol exists for measuring and validating respiratory sensitization (Golden et al. 2020). Rodent models may be the ‘gold standard’ for testing, but they are not officially approved for respiratory sensitization. Despite this, the results gained from rodent studies often fail to translate to human results (National Research Council 2007; Hartung 2008; Leist and Hartung 2013; Alves et al. 2016). Recent attempts to apply dermal tissue protocols, such as the direct peptide reactivity assay (DPRA) and peroxidase peptide reactivity assay (PPRA), to lung tissue samples have shown some positive results (Lalko et al. 2013; Dik et al. 2016). Many respiratory sensitizers may not be identifiable in the currently utilized protocols because the biomarkers that induce immune responses in the dermis differ from those in the lung. It remains challenging to identify a single standard approach using rodents (Arts 2020).

As an alternative to rodent models, human cells are increasingly popular with in vitro assays to better assess human outcomes (Perlman 2016). Studies ranging from single-cell gene testing to complex in vitro assays are currently designed to investigate multiple endpoints (Forreryd et al. 2015; Gibb and Sayes 2022). Because of the dynamic nature of the lungs (mucociliary escalator, pulmonary surfactant, changes in cell type depending on lung compartment, etc*.*), it is unclear if any specific lung region is ideal for assessing respiratory sensitizing potential. While larger particles and aerosols cannot reach the most distal regions of the lungs (*e.g.*, alveoli), this area of the lungs remains of utmost importance in the study as it is critical to gas exchange and organism survival.

This study utilized a complex, architecturally relevant, alveolar model to assess respiratory sensitization by comparing the total transcriptome after exposure to two known sensitizers (ethylene diamine and isophorone diisocyanate) and two known non-sensitizers (chlorobenzene and dimethylformamide). Comparisons were made between each known sensitizer versus non-sensitizer; further analysis included identifying the common genes between known sensitizers and non-sensitizers. Finally, significantly regulated genes were analyzed in gene ontology software designed to show which biological pathways are potentially perturbed after exposure to a known sensitizer compared to a known non-sensitizer. Previous studies have indicated that a 24-h timepoint is sufficient for cytokine and surface marker expression studies (Palmberg et al. 1998; Hellman and Eriksson 2007). Genetic expression increases can occur anywhere from 1 to 72 h after exposure (Fan et al. 1998). As such, a timepoint of 24 h for gene expression, like that of cytokine and surface marker expression, was chosen for this study. For proposed assay use, maintaining a singular timepoint for all endpoints measured expedites a potential assay and improves qualified and quantified data acquisition between laboratories.

A recent study investigating respiratory sensitizers revealed that most genes studied from a single dendritic cell assay fell within the oxidative phosphorylation and ubiquinone metabolism pathways (Forreryd et al. 2015). A multi-cell model that mimics alveolar architecture showed that the use of multiple endpoints (morphological, biochemical, genetic, and surface marker expression) increased the potential for differentiating respiratory sensitizers from a general cell activator such as phorbol 12-myristate 13 acetate (PMA) (Gibb and Sayes 2022). However, no study measures the complex in vitro model's total genetic expression. Detailed genetic information and analysis are needed to understand better respiratory sensitization from chemical xenobiotics and further the process of assay development and biomarkers of respiratory sensitization.

Gene ontology (GO) analysis was done on the totality of genetic expression for apical and basal cells, comparing sensitizers to non-sensitizers. This method allowed for identifying pathways that may lead to potential biomarker signatures for comparing known and potential sensitizers in future studies. These analyses revealed that sensitizers' cell receptor signaling, immune responses, cell cycling, and cellular organization pathways heavily regulate responses to xenobiotics compared to non-sensitizers.

Among AMs and epithelial cells, the known sensitizer ED upregulated genes associated with asthma and allergy (Himes et al. 2015; Radzikowska et al. 2022), while genes associated with T cell regulation and immune suppression (Jovanovic et al. 2011; Liu et al. 2022 were consistently downregulated. Among DCs, the known sensitizer ED upregulated genes associated with nucleosome structure, posttranscriptional modification of RNA, and airway smooth muscle contraction (Ho 2010; Yick et al. 2013; Soni and Biswas 2021). In contrast, genes associated with antigen presentation (MHCI) and mitochondrial reactive oxygen species (Viksman et al. 2002; Gao et al. 2016) were consistently downregulated.

Among AMs and epithelial cells, the known sensitizer IPDI upregulated genes associated with oxidative stress, MAPk signaling, cell adhesion and growth factors, and Wnt signaling (Paulissen et al. 2009; Carpe et al. 2012; Avasarala et al. 2013; Ejima et al. 2022). In contrast, genes associated with tRNA structure and function, suppression of severe asthma, transcription, and regulation of intracellular membrane trafficking (Salinas-Giegé et al. 2015; Takahashi et al. 2018; Cheng et al. 2022; Noguromi et al. 2023) were consistently downregulated.

Among DCs, the known sensitizer IPDI upregulated genes associated with cellular responses to stress, metabolism, membrane trafficking, transcription, and mRNA splicing (Sucre et al. 2018; Marciano et al. 2021; Domanegg et al. 2022; Faisal et al. 2022; Liang et al. 2022), while genes associated with preventing allergy, such as type I hypersensitivity and airway hyperresponsiveness, (Dharajiya et al. 2010; Übel et al. 2014) were consistently downregulated. Interestingly, regardless of which non-sensitizer was used, there were two upregulated genes among AMs and epithelial cells associated with airway hyperresponsiveness and allergy (*CCL3L1*, *GJD3*), and among DCs, there were 5 upregulated genes in common also associated with airway allergy and inflammation (*SOX9*, *UACA*, *CCDC88A*, *FOSL1*, *KIF20B*) (Laulajainen-Hongisto et al. 2020; Jiang et al. 2021; Bao and Zhu 2022).

Previous studies have attempted to characterize the transcriptome of monoculture in response to respiratory sensitizers. In human bronchial epithelial (BEAS-2B) cells, the NRF2-mediated oxidative stress response was indiscriminately activated between sensitizers and irritants, while the most discriminative genes were *BC042064*, *A_24_P229834*, *DOCK11*, *THC2544911*, *DLGAP4*, *NINJ1*, *PFKM*, *FLJ10986*, *IL28RA*, and *CASP9* (Verstraelen et al. 2009a, b, c; Remy et al. 2014). In human alveolar epithelial (A549) cells, *CTLA4* was associated with sensitization and 22 genes related to immune function (Verstraelen et al. 2009a, b, c). In human macrophages (THP-1), the most discriminating genes were related to the GO terms EIF4E, PDGFRB, SEMA7A, and ZFP36L2 (Verstraelen et al. 2009a, b, c). Further work is needed to determine which epithelial cell line is best suited to identifying respiratory sensitization and to what extent the responses of individual cells differ from when they are assembled in co-cultures.

With all current in vitro models, limitations to data interpretation and translation to humans exist. In vivo, the cellular compartments of the lungs are continually renewing, recruiting, and activating various cell types to maintain homeostasis and prevent excessive inflammation (Schilders et al. 2016; Wong et al. 2016). Cells at the gas-exchange interface, coated in cell secretions like mucus or surfactant, are exposed to air. Accordingly, many air–liquid interface (ALI) studies have shown more relevant differentiation of airway cells (*i.e.,* cilia, mucus secretion, pseudostratified epithelium) than submerged cultures (Wang et al. 2018; Silva et al. 2023). As such, it is important to consider the impact of submerged versus ALI culture systems when designing studies to investigate specific health outcomes (such as sensitization) or endpoints (such as toxicity). It is also noteworthy that the lungs secrete fluids that protect against xenobiotic materials and that these fluids should be included in models where possible or necessary. Regarding reproducibility, other known sensitizers need to be tested to verify if the panel of differentially expressed genes remains consistent or if the sensitizers have distinct classes. For now, the gene expressions and cellular effects found in this study may be compound-specific.

Further investigation is also warranted for whether this model could be extended to test for skin sensitizers. We suspect respiratory sensitization is more specific, meaning that the model performance would have less difficulty discriminating skin sensitizers. Additional work is also needed to determine if a bronchial cell model would yield similar results, whether complex mixtures are suitable for testing, or if the interactions would make the results difficult to interpret. The ability to handle mixtures is essential due to the rapid pace of new materials development.

## Conclusions

While this study and previous investigations have utilized an alveolar model, it is important to note that other designs can emulate different lung compartments or various disease states in the lungs. It is known that a respiratory sensitizer will lead to alterations in alveolar cell morphology and increases in inflammatory cytokines and surface marker expression (*e.g.,* in AMs and DCs). With several studies showing that it is possible to identify respiratory sensitizers using in vitro systems, the ability to predict and mitigate the risks of novel materials and substances is closer to realization. Such advancements are greatly needed to promote safety and sustainability across the industrial, commercial, and residential sectors.

The model used herein, in previous studies and currently, shows that differentiating sensitizers is possible (Gibb and Sayes 2022, 2023). It presents a unique opportunity to study known and novel materials for their potential to lead to long-term immune perturbations in the lungs. Using multiple modalities of investigating the transcriptome (PCA, heat maps, chord diagrams, and volcano plots) allows for easy visualization of perturbations in the gene expression across different exposure scenarios. Importantly, our analysis showed that for the sensitizers tested, the pathways associated with cell differentiation and proliferation were induced, whereas the biological processes for immune defense and functionality were inhibited. Further analysis is required to determine how these pathways may alter the immune response and how they may lead to sensitizing reactions.

## Supplementary Information

Below is the link to the electronic supplementary material.

Supplementary file1 (DOCX 126 KB)

## Data Availability

The authors declare that all data supporting the findings of this study are available within the paper, and any raw data can be obtained from the corresponding author upon request.
