# Supplementary material for: The transcriptomic signature of respiratory sensitizers using an alveolar model
Source: Cell Biol Toxicol. 2024 Apr 8;40(1):21. doi: 10.1007/s10565-024-09860-x (PMC10999393; doi:10.1007/s10565-024-09860-x)
Supplement: Supplementary file 1 — Supplementary file1 (DOCX 126 KB) [file 10565_2024_9860_MOESM1_ESM.docx]

**SUPPLEMENTAL TABLES**

Supplemental Table 1

| **Ethylenediamine: Chlorobenzene** | | | |
| --- | --- | --- | --- |
| **Down** | | **Up** | |
| ***Gene*** | **L2FC** | ***Gene*** | **L2FC** |
| *STYK1* | -2.821 | *AC019069.1* | 4.162 |
| *SLC13A2* | -2.754 | *AL137060.1* | 4.030 |
| *AC096921.2* | -2.564 | *SNAI2* | 3.162 |
| *NR2F2-AS1* | -2.532 | *CCL3L1* | 2.503 |
| *PCSK4* | -2.421 | *VANGL2* | 2.404 |
| *CD84* | -1.798 | *CCL3* | 2.257 |
| *SNAI3* | -1.751 | *CCDC65* | 2.116 |
| *IFITM1* | -1.548 | *TMEM221* | 1.871 |
| *ZFP14* | -1.444 | *N4BP3* | 1.771 |
| *TRIM22* | -1.362 | *ARSI* | 1.638 |
| *ARL11* | -1.349 | *TAGAP* | 1.352 |
| *TLE6* | -1.294 | *ALPK2* | 1.235 |
| *CR1* | -1.236 | *GJD3* | 1.232 |
| *CD101* | -1.093 | *MFSD14C* | 1.183 |
| *ZNF2* | -1.072 | *KRT6A* | 1.106 |
| *PGBD5* | -1.046 |  |  |
| *P2RX1* | -1.011 |  |  |

Supplemental Table 2

| **Ethylenediamine: Dimethylformamide** | | | |
| --- | --- | --- | --- |
| **Down** | | **Up** | |
| ***Gene*** | ***L2FC*** | ***Gene*** | **L2FC** |
| *LINC01023* | -2.999 | *RNF128* | 4.322 |
| *PLIN4* | -2.984 | *AC064836.3* | 3.585 |
| *RETN* | -2.906 | *AL928654.1* | 3.048 |
| *FABP5P7* | -2.491 | *CCL3L1* | 3.029 |
| *AC005076.1* | -2.366 | *CEACAM7* | 2.679 |
| *RHCE* | -2.047 | *HERC2P2* | 2.605 |
| *AQP8* | -2.032 | *TNF* | 2.202 |
| *RN7SL3* | -1.969 | *IL1B* | 2.178 |
| *SOWAHD* | -1.936 | *CCL3* | 2.105 |
| *FCGR2B* | -1.561 | *ZNF853* | 2.001 |
| *TMEM266* | -1.558 | *LINC00543* | 1.947 |
| *DNAJC5B* | -1.398 | *CCDC191* | 1.942 |
| *RGS18* | -1.303 | *AL162231.2* | 1.849 |
| *C1orf74* | -1.194 | *SMG1P3* | 1.802 |
| *CD101* | -1.137 | *TAGAP* | 1.749 |
| *P2RX1* | -1.129 | *AFAP1L2* | 1.394 |
| *EVI2A* | -1.122 | *ALPK2* | 1.313 |
| *ZNF232* | -1.052 | *KRT6A* | 1.288 |
| *RCBTB2* | -1.031 | *AC138969.2* | 1.277 |
| *ZNF627* | -1.028 | *GJD3* | 1.220 |
| *CD53* | -1.007 | *FAS* | 1.186 |
|  |  | *FOXQ1* | 1.173 |
|  |  | *SAMD4A* | 1.142 |
|  |  | *CCDC80* | 1.139 |
|  |  | *HEY1* | 1.103 |
|  |  | *BTG2* | 1.102 |
|  |  | *ZNF512B* | 1.088 |
|  |  | *GCC2* | 1.084 |
|  |  | *GOLGA4* | 1.084 |
|  |  | *GADD45B* | 1.070 |
|  |  | *EEA1* | 1.053 |
|  |  | *MREG* | 1.048 |
|  |  | *CENPE* | 1.036 |
|  |  | *COL27A1* | 1.034 |
|  |  | *HOXA3* | 1.023 |
|  |  | *PHACTR2* | 1.018 |
|  |  | *CCDC9B* | 1.018 |
|  |  | *HOXB3* | 1.016 |

Supplemental Table 3

| **Isophorone Diisocyanate: Chlorobenzene** | | | |
| --- | --- | --- | --- |
| **Down** | | **Up** | |
| ***Gene*** | **L2FC** | ***Gene*** | **L2FC** |
| *ONECUT1* | -3.825 | *AL034346.1* | 4.278 |
| *TGM1* | -3.056 | *PRR15L* | 3.278 |
| *MT-TE* | -2.989 | *AC135050.6* | 2.934 |
| *DLEU7* | -2.005 | *CCL3L1* | 2.259 |
| *SNAI3* | -1.963 | *RASD1* | 2.053 |
| *CCNA1* | -1.919 | *TNF* | 1.965 |
| *TSPAN2* | -1.736 | *DUSP2* | 1.766 |
| *IFITM1* | -1.582 | *GJD3* | 1.376 |
| *LINC02535* | -1.397 | *KCNV1* | 1.367 |
| *ZNF2* | -1.352 | *ADAMTS1* | 1.258 |
| *SIRPB1* | -1.314 | *AL731577.2* | 1.212 |
| *DNAJC5B* | -1.304 | *FOXQ1* | 1.106 |
| *PTPN22* | -1.299 | *KHDRBS3* | 1.057 |
| *FBXL16* | -1.279 | *CENPE* | 1.026 |
| *ARL11* | -1.277 | *FZD9* | 1.008 |
| *ZNF230* | -1.276 |  |  |
| *TLE6* | -1.196 |  |  |
| *SPRY1* | -1.136 |  |  |
| *ZNF416* | -1.090 |  |  |
| *RAB44* | -1.055 |  |  |
| *FAM78A* | -1.030 |  |  |
| *TEC* | -1.008 |  |  |

Supplemental Table 4

| **Isophorone Diisocyanate: Dimethylformamide** | | | |
| --- | --- | --- | --- |
| **Down** | | **Up** | |
| ***Gene*** | **L2FC** | ***Gene*** | **L2FC** |
| *PPP1R16B* | -3.254 | *AL391422.3* | 2.861 |
| *MT-TE* | -2.783 | *TNF* | 2.815 |
| *AC004585.1* | -2.758 | *CCL3L1* | 2.787 |
| *SNORD3A* | -2.302 | *HERC2P2* | 2.787 |
| *RNU4-2* | -2.254 | *SNHG12* | 2.669 |
| *FABP5P7* | -2.139 | *CCL3* | 2.505 |
| *DLEU7* | -1.898 | *DUSP2* | 2.446 |
| *TDRD9* | -1.839 | *RASD1* | 2.276 |
| *ZKSCAN4* | -1.660 | *PLEKHG5* | 1.927 |
| *DNAJC5B* | -1.651 | *FOXF1* | 1.894 |
| *FCGR2B* | -1.548 | *SYNGAP1* | 1.702 |
| *ZNF230* | -1.544 | *FFAR2* | 1.669 |
| *ARHGDIG* | -1.521 | *AC026401.3* | 1.662 |
| *RN7SL3* | -1.484 | *ADAMTS1* | 1.511 |
| *RGS18* | -1.313 | *TAGAP* | 1.484 |
| *TMEM266* | -1.300 | *CYP1B1-AS1* | 1.420 |
| *CCL24* | -1.234 | *GJD3* | 1.367 |
| *ZNF416* | -1.213 | *CENPE* | 1.342 |
| *RAB44* | -1.195 | *SETBP1* | 1.323 |
| *S100A12* | -1.139 | *AL731577.2* | 1.289 |
| *UQCRHL* | -1.113 | *HEY1* | 1.278 |
| *NKG7* | -1.110 | *GADD45B* | 1.238 |
| *ADGRG3* | -1.069 | *FEM1A* | 1.199 |
| *FCRLA* | -1.041 | *FRMD4B* | 1.159 |
| *SELPLG* | -1.040 | *AC240274.1* | 1.152 |
| *TXNIP* | -1.039 | *RIPK4* | 1.126 |
| *S100B* | -1.037 | *AL138724.1* | 1.126 |
| *PARVG* | -1.027 | *SEC14L4* | 1.118 |
| *FPR1* | -1.025 | *AC004854.2* | 1.087 |
|  |  | *MAP3K14* | 1.082 |
|  |  | *FZD9* | 1.076 |
|  |  | *PLD6* | 1.075 |
|  |  | *UPF3B* | 1.069 |
|  |  | *GPR183* | 1.050 |
|  |  | *NOCT* | 1.038 |
|  |  | *HOXA3* | 1.025 |
|  |  | *RND1* | 1.024 |
|  |  | *MREG* | 1.022 |

Supplemental Table 5

| **Ethylenediamine: Chlorobenzene** | | | |
| --- | --- | --- | --- |
| **Down** | | **Up** | |
| ***Gene*** | **L2FC** | ***Gene*** | **L2FC** |
| *AL390955.2* | -2.922 | *EGR3* | 6.505 |
| *HLA-DRB6* | -2.662 | *HIST1H1D* | 4.476 |
| *ZNF843* | -2.569 | *AL139099.4* | 4.476 |
| *CRB3* | -1.486 | *AKAP6* | 4.338 |
| *ZSWIM3* | -1.372 | *RNVU1-7* | 4.186 |
| *ZKSCAN4* | -1.268 | *HIST1H3B* | 4.061 |
| *P2RY12* | -1.128 | *HIST1H4A* | 4.061 |
| *ZNF816* | -1.093 | *SCARNA12* | 3.660 |
| *UQCRHL* | -1.091 | *AL355075.4* | 3.641 |
| *CCR5* | -1.090 | *HIST1H2BL* | 3.540 |
| *ZNF324* | -1.069 | *HIST2H2AB* | 3.409 |
| *RGCC* | -1.055 | *HIST1H1E* | 3.218 |
| *IFI44L* | -1.036 | *HIST1H1B* | 3.186 |
| *ZNF250* | -1.004 | *AL589880.1* | 2.955 |
|  |  | *HIST1H2AE* | 2.955 |
|  |  | *SCARNA7* | 2.732 |
|  |  | *AC012073.1* | 2.660 |
|  |  | *HIST2H3D* | 2.649 |
|  |  | *HIST1H2BE* | 2.519 |
|  |  | *AC116050.1* | 2.253 |
|  |  | *CNTN6* | 2.218 |
|  |  | *SCARF2* | 2.121 |
|  |  | *LINC00294* | 2.111 |
|  |  | *HIST1H4E* | 2.097 |
|  |  | *HIST2H2BF* | 2.058 |
|  |  | *IL10* | 1.995 |
|  |  | *CACNG4* | 1.964 |
|  |  | *RNU4-1* | 1.853 |
|  |  | *WASH2P* | 1.837 |
|  |  | *ACKR3* | 1.706 |
|  |  | *SCAPER* | 1.658 |
|  |  | *NFIB* | 1.601 |
|  |  | *SOX9* | 1.584 |
|  |  | *LINGO1* | 1.557 |
|  |  | *GCC2* | 1.468 |
|  |  | *CENPF* | 1.444 |
|  |  | *ARID4A* | 1.407 |
|  |  | *UACA* | 1.342 |
|  |  | *NR4A3* | 1.338 |
|  |  | *LMTK3* | 1.316 |
|  |  | *CCDC88A* | 1.262 |
|  |  | *GEM* | 1.252 |
|  |  | *CCDC170* | 1.242 |
|  |  | *AC245041.1* | 1.211 |
|  |  | *EEA1* | 1.209 |
|  |  | *GADD45B* | 1.208 |
|  |  | *RND1* | 1.188 |
|  |  | *CEP350* | 1.173 |
|  |  | *MPHOSPH8* | 1.150 |
|  |  | *PIBF1* | 1.150 |
|  |  | *UPF2* | 1.138 |
|  |  | *GLIS2* | 1.111 |
|  |  | *IGF2BP2* | 1.099 |
|  |  | *TPR* | 1.092 |
|  |  | *MAP7D3* | 1.088 |
|  |  | *KTN1* | 1.080 |
|  |  | *FOSL1* | 1.055 |
|  |  | *KIF20B* | 1.053 |
|  |  | *MTSS1L* | 1.050 |
|  |  | *GJC1* | 1.049 |
|  |  | *ZC3H13* | 1.016 |
|  |  | *GOLGA8N* | 1.016 |
|  |  | *CWF19L2* | 1.008 |
|  |  | *ZSWIM9* | 1.008 |
|  |  | *ARHGAP21* | 1.007 |

Supplemental Table 6

| **Ethylenediamine: Dimethylformamide** | | | |
| --- | --- | --- | --- |
| **Down** | | **Up** | |
| ***Gene*** | **L2FC** | ***Gene*** | **L2FC** |
| *ARL17B* | -2.952 | *HIST1H1E* | 5.479 |
| *MT-TT* | -2.192 | *SCARNA7* | 5.479 |
| *BRF2* | -1.539 | *HIST1H1B* | 5.125 |
| *TBC1D19* | -1.520 | *HIST1H4A* | 5.000 |
| *HLA-G* | -1.432 | *TNFSF18* | 4.909 |
| *OR52K3P* | -1.406 | *HIST2H3D* | 4.909 |
| *TDRD6* | -1.343 | *AL139099.4* | 4.415 |
| *GPR65* | -1.335 | *AL031777.3* | 4.348 |
| *NHLRC1* | -1.334 | *HIST1H2BE* | 4.043 |
| *TXNIP* | -1.332 | *EGR4* | 3.894 |
| *ATP6V0D2* | -1.219 | *RNU4-1* | 3.862 |
| *RXFP1* | -1.210 | *HIST1H4L* | 3.710 |
| *VSIG4* | -1.191 | *HIST1H2AH* | 3.656 |
| *ZNF627* | -1.154 | *SCARNA12* | 3.599 |
| *ZNF322* | -1.147 | *SLC7A5P1* | 3.540 |
| *SNAI3* | -1.116 | *HIST1H2BC* | 3.370 |
| *ZNF695* | -1.106 | *HIST2H2AB* | 3.348 |
| *ADORA3* | -1.106 | *ZFHX4* | 3.324 |
| *MT1E* | -1.099 | *HIST1H2BN* | 2.919 |
| *ZNF845* | -1.080 | *CGN* | 2.909 |
| *RSAD2* | -1.070 | *ADAMTS9* | 2.881 |
| *C1orf74* | -1.067 | *LOXL4* | 2.763 |
| *IFI27L2* | -1.067 | *TCAF1P1* | 2.656 |
| *CLK1* | -1.052 | *HIST2H2BF* | 2.625 |
| *ZNF691* | -1.043 | *KAT6B* | 2.618 |
| *UQCRHL* | -1.035 | *CEP126* | 2.470 |
| *GPR34* | -1.032 | *COL11A1* | 2.313 |
| *USP9Y* | -1.021 | *CENPE* | 2.306 |
| *TRIM22* | -1.010 | *COL27A1* | 2.248 |
| *ZNF586* | -1.001 | *SOX9* | 2.217 |
|  |  | *SCARF2* | 2.212 |
|  |  | *CENPF* | 2.211 |
|  |  | *HIST1H4E* | 2.152 |
|  |  | *HOXA3* | 2.114 |
|  |  | *UACA* | 2.109 |
|  |  | *RBFOX3* | 2.100 |
|  |  | *GCC2* | 2.091 |
|  |  | *HIST1H2AI* | 2.050 |
|  |  | *LINC00294* | 2.050 |
|  |  | *LRRN3* | 2.026 |
|  |  | *RASGEF1B* | 2.012 |
|  |  | *TAF3* | 1.989 |
|  |  | *EIF5B* | 1.952 |
|  |  | *ENTPD2* | 1.906 |
|  |  | *TEAD3* | 1.868 |
|  |  | *ZNF37A* | 1.868 |
|  |  | *SUSD2* | 1.866 |
|  |  | *KIF20B* | 1.802 |
|  |  | *NLGN2* | 1.763 |
|  |  | *CTGF* | 1.740 |
|  |  | *KIF15* | 1.740 |
|  |  | *CXCL2* | 1.731 |
|  |  | *GOLGA3* | 1.721 |
|  |  | *HIC1* | 1.698 |
|  |  | *ZC3H12A* | 1.679 |
|  |  | *ARHGAP21* | 1.667 |
|  |  | *TPR* | 1.657 |
|  |  | *TCOF1* | 1.641 |
|  |  | *CDC42EP2* | 1.636 |
|  |  | *ECE1* | 1.634 |
|  |  | *MTX1P1* | 1.618 |
|  |  | *UHRF1* | 1.613 |
|  |  | *PLEKHA6* | 1.609 |
|  |  | *FYCO1* | 1.605 |
|  |  | *MAP9* | 1.599 |
|  |  | *RAD50* | 1.594 |
|  |  | *MAP1B* | 1.585 |
|  |  | *NUAK2* | 1.569 |
|  |  | *THBS1* | 1.559 |
|  |  | *CYR61* | 1.554 |
|  |  | *RIPOR1* | 1.551 |
|  |  | *PCSK1N* | 1.549 |
|  |  | *CASKIN2* | 1.536 |
|  |  | *SLC16A13* | 1.528 |
|  |  | *BPIFB1* | 1.525 |
|  |  | *NCOR1* | 1.523 |
|  |  | *LIMCH1* | 1.507 |
|  |  | *PPP1R15A* | 1.495 |
|  |  | *CD3EAP* | 1.495 |
|  |  | *SPTBN1* | 1.489 |
|  |  | *CCDC102A* | 1.482 |
|  |  | *CHGB* | 1.474 |
|  |  | *PPP1R9A* | 1.474 |
|  |  | *GLIS2* | 1.473 |
|  |  | *SLK* | 1.471 |
|  |  | *TNFAIP8L1* | 1.470 |
|  |  | *FOSL1* | 1.469 |
|  |  | *CCDC88A* | 1.459 |
|  |  | *MAML3* | 1.446 |
|  |  | *MINK1* | 1.429 |
|  |  | *PIBF1* | 1.428 |
|  |  | *GRIPAP1* | 1.421 |
|  |  | *BCAM* | 1.416 |
|  |  | *CADM4* | 1.411 |
|  |  | *ATRX* | 1.411 |
|  |  | *DOCK9* | 1.409 |
|  |  | *C1R* | 1.408 |
|  |  | *RND3* | 1.407 |
|  |  | *CLIP1* | 1.403 |
|  |  | *ARID4B* | 1.401 |
|  |  | *C3* | 1.401 |
|  |  | *ZC3H4* | 1.401 |
|  |  | *PRR11* | 1.394 |
|  |  | *PHLDA1* | 1.388 |
|  |  | *KRT6A* | 1.384 |
|  |  | *EVPL* | 1.382 |
|  |  | *KTN1* | 1.382 |
|  |  | *ROCK2* | 1.374 |
|  |  | *DYRK1B* | 1.361 |
|  |  | *TGFB1I1* | 1.355 |
|  |  | *ACKR3* | 1.352 |
|  |  | *MYH9* | 1.350 |
|  |  | *WWC1* | 1.341 |
|  |  | *CAVIN2* | 1.340 |
|  |  | *MAP4* | 1.338 |
|  |  | *ZNF609* | 1.338 |
|  |  | *GPRIN1* | 1.338 |
|  |  | *SETD1A* | 1.338 |
|  |  | *MPHOSPH8* | 1.328 |
|  |  | *AMOTL2* | 1.326 |
|  |  | *TBX3* | 1.322 |
|  |  | *AFDN* | 1.321 |
|  |  | *CDC42BPA* | 1.321 |
|  |  | *RRBP1* | 1.319 |
|  |  | *SMARCC2* | 1.313 |
|  |  | *TCF20* | 1.313 |
|  |  | *CNNM1* | 1.312 |
|  |  | *MYO10* | 1.310 |
|  |  | *CAST* | 1.308 |
|  |  | *IER2* | 1.302 |
|  |  | *THRAP3* | 1.302 |
|  |  | *BBX* | 1.300 |
|  |  | *LARP1* | 1.298 |
|  |  | *CDR2L* | 1.295 |
|  |  | *ACTN4* | 1.294 |
|  |  | *SRRM1* | 1.293 |
|  |  | *CSRNP1* | 1.292 |
|  |  | *DNMT1* | 1.292 |
|  |  | *CEP350* | 1.289 |
|  |  | *NRP2* | 1.285 |
|  |  | *MYO15B* | 1.285 |
|  |  | *FILIP1* | 1.285 |
|  |  | *ZNF628* | 1.277 |
|  |  | *TFAP2A* | 1.273 |
|  |  | *NCKAP5L* | 1.272 |
|  |  | *COL7A1* | 1.271 |
|  |  | *NSRP1* | 1.271 |
|  |  | *KIF16B* | 1.270 |
|  |  | *RAI14* | 1.267 |
|  |  | *JUNB* | 1.263 |
|  |  | *MIDN* | 1.261 |
|  |  | *CHD3* | 1.258 |
|  |  | *RFC1* | 1.258 |
|  |  | *RILPL1* | 1.256 |
|  |  | *CEACAM5* | 1.252 |
|  |  | *MTCL1* | 1.251 |
|  |  | *EPB41L1* | 1.249 |
|  |  | *SEMA3B* | 1.249 |
|  |  | *BICD2* | 1.244 |
|  |  | *TCF7L2* | 1.243 |
|  |  | *SOX12* | 1.241 |
|  |  | *TRIP11* | 1.239 |
|  |  | *PTPN14* | 1.237 |
|  |  | *SOCS3* | 1.236 |
|  |  | *IGF1R* | 1.235 |
|  |  | *HLX* | 1.234 |
|  |  | *CALD1* | 1.234 |
|  |  | *ARID4A* | 1.233 |
|  |  | *TTLL5* | 1.233 |
|  |  | *TRAF3IP1* | 1.233 |
|  |  | *MAP3K10* | 1.233 |
|  |  | *EIF4G3* | 1.231 |
|  |  | *HNRNPUL2* | 1.229 |
|  |  | *CKAP5* | 1.228 |
|  |  | *DST* | 1.227 |
|  |  | *IER5* | 1.224 |
|  |  | *NUMA1* | 1.221 |
|  |  | *PLK2* | 1.219 |
|  |  | *KIF18B* | 1.218 |
|  |  | *ROBO1* | 1.217 |
|  |  | *COL18A1* | 1.216 |
|  |  | *MYBBP1A* | 1.216 |
|  |  | *PHF3* | 1.215 |
|  |  | *KNL1* | 1.214 |
|  |  | *RAB3B* | 1.214 |
|  |  | *CAVIN1* | 1.214 |
|  |  | *ARHGEF18* | 1.210 |
|  |  | *ELMSAN1* | 1.202 |
|  |  | *TJP1* | 1.200 |
|  |  | *MAP7D1* | 1.199 |
|  |  | *CDK11B* | 1.195 |
|  |  | *CLCF1* | 1.194 |
|  |  | *PHF21A* | 1.190 |
|  |  | *EHD2* | 1.186 |
|  |  | *MYO18A* | 1.185 |
|  |  | *UTP14A* | 1.185 |
|  |  | *GPATCH1* | 1.184 |
|  |  | *BCL6* | 1.181 |
|  |  | *CHD6* | 1.178 |
|  |  | *COL5A2* | 1.177 |
|  |  | *ZC3H13* | 1.177 |
|  |  | *COL4A2* | 1.177 |
|  |  | *CDCP1* | 1.173 |
|  |  | *ABCF1* | 1.172 |
|  |  | *TNKS1BP1* | 1.171 |
|  |  | *PHLDB2* | 1.169 |
|  |  | *PTGER4* | 1.166 |
|  |  | *TOX2* | 1.166 |
|  |  | *RAI1* | 1.161 |
|  |  | *SGO2* | 1.161 |
|  |  | *MGAT5B* | 1.160 |
|  |  | *PPP1R12A* | 1.160 |
|  |  | *CDH1* | 1.160 |
|  |  | *MYC* | 1.160 |
|  |  | *BAZ1B* | 1.159 |
|  |  | *MYO9B* | 1.158 |
|  |  | *BHLHE40* | 1.157 |
|  |  | *BAZ1A* | 1.157 |
|  |  | *KCNJ2* | 1.149 |
|  |  | *CIR1* | 1.147 |
|  |  | *DPYSL2* | 1.146 |
|  |  | *MKI67* | 1.145 |
|  |  | *DBNDD1* | 1.145 |
|  |  | *TNS4* | 1.145 |
|  |  | *LARP7* | 1.142 |
|  |  | *TUBB4A* | 1.141 |
|  |  | *SAFB2* | 1.139 |
|  |  | *CRIM1* | 1.138 |
|  |  | *SCAF1* | 1.137 |
|  |  | *CLU* | 1.137 |
|  |  | *MYH10* | 1.137 |
|  |  | *PRRC2C* | 1.131 |
|  |  | *ABL1* | 1.130 |
|  |  | *SETD2* | 1.129 |
|  |  | *CIT* | 1.122 |
|  |  | *TRIM8* | 1.120 |
|  |  | *LAMA5* | 1.117 |
|  |  | *PMAIP1* | 1.116 |
|  |  | *NOLC1* | 1.116 |
|  |  | *DUSP1* | 1.114 |
|  |  | *RHOB* | 1.112 |
|  |  | *LSR* | 1.112 |
|  |  | *GADD45B* | 1.110 |
|  |  | *MYH14* | 1.109 |
|  |  | *PRPF4B* | 1.107 |
|  |  | *TACC2* | 1.104 |
|  |  | *PLCH1* | 1.097 |
|  |  | *WFS1* | 1.096 |
|  |  | *DNM1* | 1.091 |
|  |  | *GATAD2A* | 1.087 |
|  |  | *CELSR1* | 1.085 |
|  |  | *SMC2* | 1.085 |
|  |  | *PRRC2B* | 1.081 |
|  |  | *ARID1A* | 1.077 |
|  |  | *RTN4RL2* | 1.077 |
|  |  | *SHROOM3* | 1.075 |
|  |  | *TCERG1* | 1.072 |
|  |  | *CSF1* | 1.071 |
|  |  | *ITSN1* | 1.069 |
|  |  | *RSF1* | 1.069 |
|  |  | *ARHGAP23* | 1.067 |
|  |  | *ZFP36L1* | 1.066 |
|  |  | *FLNA* | 1.065 |
|  |  | *HNRNPU* | 1.064 |
|  |  | *NOTCH3* | 1.064 |
|  |  | *EPHB4* | 1.061 |
|  |  | *SMARCA4* | 1.060 |
|  |  | *SMC3* | 1.054 |
|  |  | *ERBB2* | 1.053 |
|  |  | *IGF2BP2* | 1.052 |
|  |  | *KIF1C* | 1.051 |
|  |  | *RUSC2* | 1.051 |
|  |  | *CEP164* | 1.050 |
|  |  | *SART1* | 1.048 |
|  |  | *XYLT2* | 1.043 |
|  |  | *MTR* | 1.040 |
|  |  | *PNN* | 1.038 |
|  |  | *DSP* | 1.038 |
|  |  | *UBALD1* | 1.038 |
|  |  | *ARID1B* | 1.038 |
|  |  | *ATN1* | 1.036 |
|  |  | *KDM4B* | 1.030 |
|  |  | *SMC6* | 1.030 |
|  |  | *MARK2* | 1.030 |
|  |  | *SMC4* | 1.029 |
|  |  | *NFIC* | 1.028 |
|  |  | *G6PD* | 1.027 |
|  |  | *CTTN* | 1.026 |
|  |  | *TAF15* | 1.025 |
|  |  | *SERPINE1* | 1.023 |
|  |  | *KRI1* | 1.020 |
|  |  | *FMNL1* | 1.018 |
|  |  | *ZNF185* | 1.016 |
|  |  | *SREBF1* | 1.016 |
|  |  | *ITPR3* | 1.016 |
|  |  | *LGALS3BP* | 1.014 |
|  |  | *CHD1* | 1.011 |
|  |  | *AGRN* | 1.010 |
|  |  | *MEX3D* | 1.010 |
|  |  | *NIN* | 1.008 |
|  |  | *PPP1R16A* | 1.006 |
|  |  | *ITGB4* | 1.004 |
|  |  | *LRFN3* | 1.004 |
|  |  | *SGK1* | 1.003 |
|  |  | *BICC1* | 1.001 |

Supplemental Table 7

| **Isophorone Diisocyanate: Chlorobenzene** | | | |
| --- | --- | --- | --- |
| **Down** | | **Up** | |
| ***Gene*** | **L2FC** | ***Gene*** | **L2FC** |
| *RNU4-1* | -3.066 | *EGR3* | 6.335 |
| *ARMC10P1* | -2.891 | *AC069544.1* | 2.800 |
| *TRGV7* | -2.359 | *KANSL1-AS1* | 2.628 |
| *ZNF98* | -2.259 | *IL10* | 2.268 |
| *RNU4-2* | -2.229 | *LRRC37A3* | 1.982 |
| *SPATA9* | -2.166 | *NA* | 1.620 |
| *AC008568.1* | -2.067 | *SOX9* | 1.596 |
| *PLAG1* | -1.894 | *ACKR3* | 1.527 |
| *SPRY3* | -1.844 | *ADRB2* | 1.498 |
| *FAM212A* | -1.829 | *CEP152* | 1.467 |
| *ZNF416* | -1.600 | *LMTK3* | 1.386 |
| *ZNF449* | -1.556 | *NFIB* | 1.386 |
| *SIT1* | -1.390 | *AC125257.1* | 1.355 |
| *ZNF2* | -1.386 | *NR4A3* | 1.283 |
| *CCR5* | -1.294 | *EEA1* | 1.255 |
| *ABALON* | -1.290 | *SGO2* | 1.245 |
| *ARHGAP11B* | -1.279 | *GEM* | 1.225 |
| *ZNF773* | -1.259 | *MAP7D3* | 1.207 |
| *ZNF671* | -1.229 | *CEMIP* | 1.203 |
| *AC140134.1* | -1.228 | *RND1* | 1.194 |
| *GRIK2* | -1.226 | *UACA* | 1.176 |
| *ZNF230* | -1.203 | *AXIN2* | 1.166 |
| *ZNF816* | -1.179 | *CCDC88A* | 1.131 |
| *ZKSCAN4* | -1.157 | *OFD1* | 1.126 |
| *FCGR2B* | -1.095 | *DNAJC2* | 1.113 |
| *ZNF250* | -1.072 | *ARID4A* | 1.086 |
| *ATP6V0D2* | -1.062 | *NR2F1* | 1.058 |
| *HCST* | -1.060 | *KIF20B* | 1.056 |
| *IL31RA* | -1.058 | *FOSL1* | 1.044 |
| *BATF2* | -1.031 | *ZNRF1* | 1.030 |
| *AP001099.1* | -1.026 | *NSRP1* | 1.024 |
| *PARS2* | -1.025 | *GATA2* | 1.021 |
| *P2RY12* | -1.008 | *KIF21A* | 1.012 |
| *ZNF566* | -1.006 |  |  |

Supplemental Table 8

| **Isophorone Diisocyanate: Dimethylformamide** | | | |
| --- | --- | --- | --- |
| **Down** | | **Up** | |
| ***Gene*** | **L2FC** | ***Gene*** | **L2FC** |
| *AC114956.3* | -2.772 | *TNFSF18* | 5.211 |
| *RNASEH2B-AS1* | -2.691 | *PLEKHN1* | 3.594 |
| *PLAG1* | -2.087 | *AC124798.1* | 3.272 |
| *ZNF10* | -2.058 | *ADAMTS9* | 3.009 |
| *LINC01169* | -1.868 | *DEGS2* | 2.731 |
| *ZNF416* | -1.787 | *RNF208* | 2.687 |
| *ZNF449* | -1.707 | *AMH* | 2.641 |
| *ZNF420* | -1.576 | *RASD1* | 2.641 |
| *KIT* | -1.563 | *HIST1H2BC* | 2.424 |
| *FAM212A* | -1.542 | *LRRC37A3* | 2.387 |
| *ZNF230* | -1.466 | *ATAD5* | 2.292 |
| *ZNF606* | -1.397 | *SOX9* | 2.236 |
| *ATP6V0D2* | -1.346 | *MSX1* | 2.228 |
| *ZKSCAN8* | -1.326 | *PCSK1N* | 2.009 |
| *FPR3* | -1.313 | *KAT6B* | 1.970 |
| *AC098613.1* | -1.294 | *UACA* | 1.950 |
| *FCGR2B* | -1.284 | *CEP152* | 1.943 |
| *ZNF627* | -1.283 | *HOXA5* | 1.943 |
| *ZNF816* | -1.268 | *AP003392.4* | 1.883 |
| *NHLRC1* | -1.261 | *HOXA3* | 1.825 |
| *ZNF549* | -1.249 | *KIF20B* | 1.813 |
| *ALDOC* | -1.245 | *LRRN3* | 1.780 |
| *ZNF671* | -1.241 | *FBLN1* | 1.760 |
| *BATF2* | -1.218 | *TEAD3* | 1.708 |
| *ZNF322* | -1.217 | *CEMIP* | 1.707 |
| *GPR65* | -1.193 | *RBFOX3* | 1.706 |
| *VSIG4* | -1.171 | *KIF15* | 1.700 |
| *PARS2* | -1.164 | *FYCO1* | 1.699 |
| *ADORA3* | -1.144 | *EIF5B* | 1.674 |
| *GPR34* | -1.144 | *TAF3* | 1.670 |
| *BRF2* | -1.140 | *SUSD2* | 1.663 |
| *C1orf74* | -1.134 | *CENPF* | 1.632 |
| *ZC3H10* | -1.105 | *SOX21-AS1* | 1.603 |
| *TRDN* | -1.105 | *CTGF* | 1.599 |
| *USP9Y* | -1.100 | *AFDN* | 1.587 |
| *AC138761.1* | -1.093 | *FOXF2* | 1.562 |
| *ZNF436* | -1.089 | *SIK1B* | 1.561 |
| *GPR141* | -1.087 | *ATRX* | 1.542 |
| *TP53INP1* | -1.043 | *PCSK9* | 1.541 |
| *GIMAP6* | -1.026 | *SPIRE2* | 1.525 |
| *ZNF691* | -1.023 | *ZC3H12A* | 1.515 |
| *CMKLR1* | -1.022 | *HNF1A* | 1.511 |
| *ZNF561* | -1.010 | *UHRF1* | 1.510 |
|  |  | *PPP1R9A* | 1.500 |
|  |  | *SEMA3B* | 1.474 |
|  |  | *FOSL1* | 1.464 |
|  |  | *NUAK2* | 1.454 |
|  |  | *CCDC102A* | 1.434 |
|  |  | *NA* | 1.432 |
|  |  | *CD3EAP* | 1.432 |
|  |  | *GPRIN1* | 1.417 |
|  |  | *SPATA33* | 1.412 |
|  |  | *ECE1* | 1.412 |
|  |  | *PRR11* | 1.401 |
|  |  | *TNFAIP8L1* | 1.393 |
|  |  | *SRRM1* | 1.385 |
|  |  | *NSRP1* | 1.385 |
|  |  | *CDR2L* | 1.383 |
|  |  | *PLEKHA6* | 1.381 |
|  |  | *EVPL* | 1.378 |
|  |  | *OSBPL7* | 1.371 |
|  |  | *CEACAM5* | 1.368 |
|  |  | *PMAIP1* | 1.365 |
|  |  | *C1R* | 1.364 |
|  |  | *MAP7D3* | 1.363 |
|  |  | *NLGN2* | 1.358 |
|  |  | *RAD50* | 1.353 |
|  |  | *ROCK2* | 1.351 |
|  |  | *TCOF1* | 1.340 |
|  |  | *BBX* | 1.336 |
|  |  | *CCDC88A* | 1.335 |
|  |  | *HIC1* | 1.329 |
|  |  | *DNMT1* | 1.324 |
|  |  | *FILIP1* | 1.317 |
|  |  | *KNL1* | 1.311 |
|  |  | *AKAP12* | 1.302 |
|  |  | *CYR61* | 1.300 |
|  |  | *FEM1A* | 1.297 |
|  |  | *RADIL* | 1.297 |
|  |  | *LIMCH1* | 1.290 |
|  |  | *TPR* | 1.286 |
|  |  | *FAM155B* | 1.283 |
|  |  | *CLIP1* | 1.280 |
|  |  | *MIDN* | 1.278 |
|  |  | *MAP1B* | 1.277 |
|  |  | *LRFN1* | 1.272 |
|  |  | *CASKIN2* | 1.268 |
|  |  | *LSR* | 1.265 |
|  |  | *CAST* | 1.264 |
|  |  | *GRIPAP1* | 1.258 |
|  |  | *PHLDA1* | 1.258 |
|  |  | *DZIP1* | 1.255 |
|  |  | *MYC* | 1.250 |
|  |  | *MYBBP1A* | 1.247 |
|  |  | *KTN1* | 1.244 |
|  |  | *SOCS3* | 1.243 |
|  |  | *SLK* | 1.242 |
|  |  | *ZFPM1* | 1.218 |
|  |  | *MAP3K10* | 1.213 |
|  |  | *NR2F1* | 1.207 |
|  |  | *AMOTL2* | 1.204 |
|  |  | *RND3* | 1.202 |
|  |  | *CKAP5* | 1.197 |
|  |  | *CIR1* | 1.192 |
|  |  | *SLC9A3R2* | 1.182 |
|  |  | *TNS4* | 1.178 |
|  |  | *MEX3D* | 1.178 |
|  |  | *AXIN2* | 1.176 |
|  |  | *RFC1* | 1.172 |
|  |  | *GADD45B* | 1.172 |
|  |  | *GATA2* | 1.170 |
|  |  | *TRAF3IP1* | 1.170 |
|  |  | *MPHOSPH8* | 1.166 |
|  |  | *CADM4* | 1.165 |
|  |  | *TNS2* | 1.164 |
|  |  | *CLU* | 1.155 |
|  |  | *RHOB* | 1.154 |
|  |  | *CTXN1* | 1.151 |
|  |  | *SMARCA4* | 1.147 |
|  |  | *NCOR1* | 1.146 |
|  |  | *COL18A1* | 1.143 |
|  |  | *LGALS3BP* | 1.142 |
|  |  | *CSPP1* | 1.142 |
|  |  | *TFAP2A* | 1.141 |
|  |  | *UTP14A* | 1.140 |
|  |  | *KIF18B* | 1.139 |
|  |  | *HLX* | 1.139 |
|  |  | *DNAJC2* | 1.134 |
|  |  | *ARHGAP23* | 1.130 |
|  |  | *PTGER4* | 1.128 |
|  |  | *ESF1* | 1.128 |
|  |  | *WFS1* | 1.127 |
|  |  | *SREBF1* | 1.126 |
|  |  | *CDC42BPA* | 1.123 |
|  |  | *GLIS2* | 1.122 |
|  |  | *KIF21A* | 1.121 |
|  |  | *SAFB2* | 1.118 |
|  |  | *SPTBN1* | 1.116 |
|  |  | *NIN* | 1.116 |
|  |  | *WWC1* | 1.115 |
|  |  | *SART1* | 1.113 |
|  |  | *KCNJ2* | 1.110 |
|  |  | *PPP1R16A* | 1.107 |
|  |  | *BCAM* | 1.107 |
|  |  | *ABCF1* | 1.104 |
|  |  | *SCEL* | 1.102 |
|  |  | *C3* | 1.098 |
|  |  | *RAI1* | 1.097 |
|  |  | *BCL3* | 1.092 |
|  |  | *BHLHE40* | 1.091 |
|  |  | *TAGAP* | 1.090 |
|  |  | *SOX13* | 1.090 |
|  |  | *WSCD1* | 1.090 |
|  |  | *CHGB* | 1.083 |
|  |  | *MYO15B* | 1.082 |
|  |  | *GPATCH4* | 1.081 |
|  |  | *RRBP1* | 1.079 |
|  |  | *DNM1* | 1.078 |
|  |  | *KRI1* | 1.070 |
|  |  | *NUMBL* | 1.063 |
|  |  | *PLEKHH3* | 1.063 |
|  |  | *CALD1* | 1.063 |
|  |  | *TJP1* | 1.062 |
|  |  | *PLK2* | 1.055 |
|  |  | *MICALL1* | 1.055 |
|  |  | *BAZ1A* | 1.054 |
|  |  | *HBEGF* | 1.053 |
|  |  | *ZNF628* | 1.052 |
|  |  | *ARHGAP21* | 1.050 |
|  |  | *NUMA1* | 1.048 |
|  |  | *COL4A2* | 1.047 |
|  |  | *MPDZ* | 1.045 |
|  |  | *MINK1* | 1.043 |
|  |  | *TUBB4A* | 1.043 |
|  |  | *PRR7* | 1.043 |
|  |  | *CTTN* | 1.042 |
|  |  | *PLCH1* | 1.040 |
|  |  | *MGAT5B* | 1.037 |
|  |  | *LRFN3* | 1.036 |
|  |  | *ZNF865* | 1.035 |
|  |  | *DSP* | 1.034 |
|  |  | *NOLC1* | 1.030 |
|  |  | *GTF2IRD1* | 1.030 |
|  |  | *JAG2* | 1.029 |
|  |  | *COL4A5* | 1.028 |
|  |  | *COL5A2* | 1.025 |
|  |  | *FARP1* | 1.025 |
|  |  | *GPATCH1* | 1.024 |
|  |  | *SMC6* | 1.023 |
|  |  | *ZNF219* | 1.022 |
|  |  | *BCL6* | 1.018 |
|  |  | *SMARCC2* | 1.016 |
|  |  | *IGFBP1* | 1.016 |
|  |  | *CST1* | 1.014 |
|  |  | *FSTL4* | 1.012 |
|  |  | *ASAP2* | 1.012 |
|  |  | *KIF4A* | 1.011 |
|  |  | *CNOT3* | 1.010 |
|  |  | *SMC3* | 1.010 |
|  |  | *MFGE8* | 1.009 |
|  |  | *TCAF1* | 1.009 |
|  |  | *CDK11B* | 1.008 |
|  |  | *DUSP1* | 1.005 |
|  |  | *SCAF1* | 1.005 |
|  |  | *CAVIN1* | 1.004 |
|  |  | *PPP1R12A* | 1.002 |
|  |  | *CHD3* | 1.001 |

Supplemental Table 9.
